# Supplementary material for: Conditional deep learning model reveals translation elongation determinants during amino acid deprivation
Source: Commun Biol. 2025 Nov 26;8:1691. doi: 10.1038/s42003-025-09092-7 (PMC12657957; doi:10.1038/s42003-025-09092-7)
Supplement: Supplementary file 2 — Supplementary [file 42003_2025_9092_MOESM2_ESM.pdf]

Conditional Deep Learning Model Reveals Translation Elongation Determinants during Amino Acid Deprivation

Mohan Vamsi Nallapareddy[1†], Francesco Craighero[1†], Lina Worpenberg[2], Felix Naef[2], Cédric Gobet[2*], Pierre Vandergheynst[1*]

[1]LTS2 Signal Processing Laboratory, IEM, STI, École Polytechnique Fédérale de Lausanne, Rte Cantonale, Lausanne, 1015, Vaud, Switzerland.
[2]UPNAE Laboratory of Computational and Systems Biology, IBI, SV, École Polytechnique Fédérale de Lausanne, Rte Cantonale, Lausanne, 1015, Vaud, Switzerland.

*Corresponding author(s). E-mail(s): cédric.gobet@epfl.ch; pierre.vandergheynst@epfl.ch;
Contributing authors: vamsi.nallapareddy@epfl.ch; francesco.craighero@epfl.ch; lina.worpenberg@epfl.ch; felix.naef@epfl.ch;
†These authors contributed equally to this work.

Supplementary Figures and Tables

Method	Year	Features	Label Corr.	Out	Predictor
riboShape [1]	2016	ctx	denoise	cdn	LM
RUST [2]	2016	ctx	quant	cdn	CS
ROSE [3]	2017	ctx	quant	cdn	CNN
Iχnos [4]	2018	ctx, fold	norm	cdn	FNN
DeepShape [5]	2019	ctx	norm	cdn	CNN
Riboexp [6]	2021	ctx, fold	norm	cdn	RNN
RiboMIMO [7]	2021	CDS, fold	norm, quant	seq	RNN
RiboGL [8]	2024	seq, 2struct	norm	seq	RNN+GNN
Riboformer [9]	2024	ctx	norm	ctx	MHA
Translatomer [10]	2024	seq, expr	norm	seq	MHA
EIF [11]	2024	As	norm	cdn	iForest
Riboclette (ours)	2024	seq, depr	norm	seq	MHA

Table S1: **Summary of ribosome density modeling approaches.** Features: A-site counts and position (As), codon, nucleotide, and/or amino acid of the context surrounding the A/P-site (ctx) or the whole sequence (seq), mRNA folding energies (fold), amino acid deprivation context (depr), mRNA secondary structure (2struct), RNA expression (expr). Label Correction: denoising (denoise), quantization (quant), and normalization, e.g., dividing by average transcript density (norm). Output: at codon (cdn), context (ctx) or sequence (seq) level. Predictor: Codon-wise Statistics (CS), Linear Model (LM), Feedforward, Convolutional, Graph and Recurrent Neural Network (FNN, CNN, GNN, and RNN, respectively), Transformers with Multi-Head Attention (MHA), and Isolation Forests (iForest).

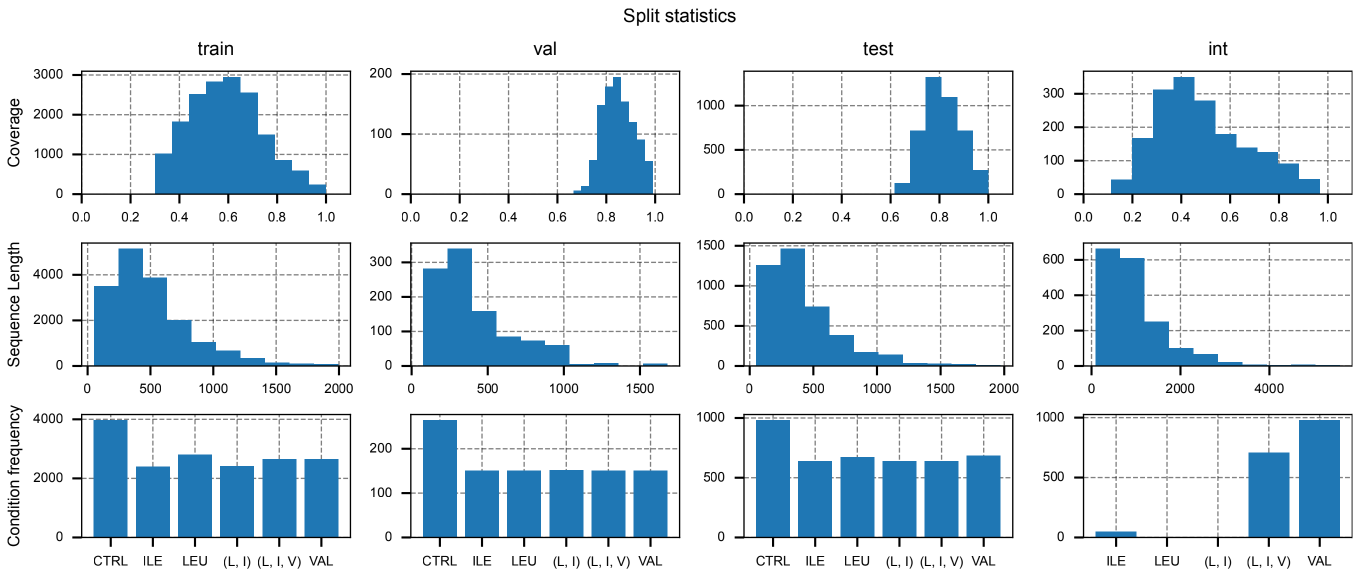

Fig. S1: **Statistics of each data split.** For the $\mathcal{D}^{train}$, $\mathcal{D}^{val}$, $\mathcal{D}^{test}$, and $\mathcal{D}^{int}$ splits, we reported the coverage and sequence length distribution and the frequency of each deprivation condition.

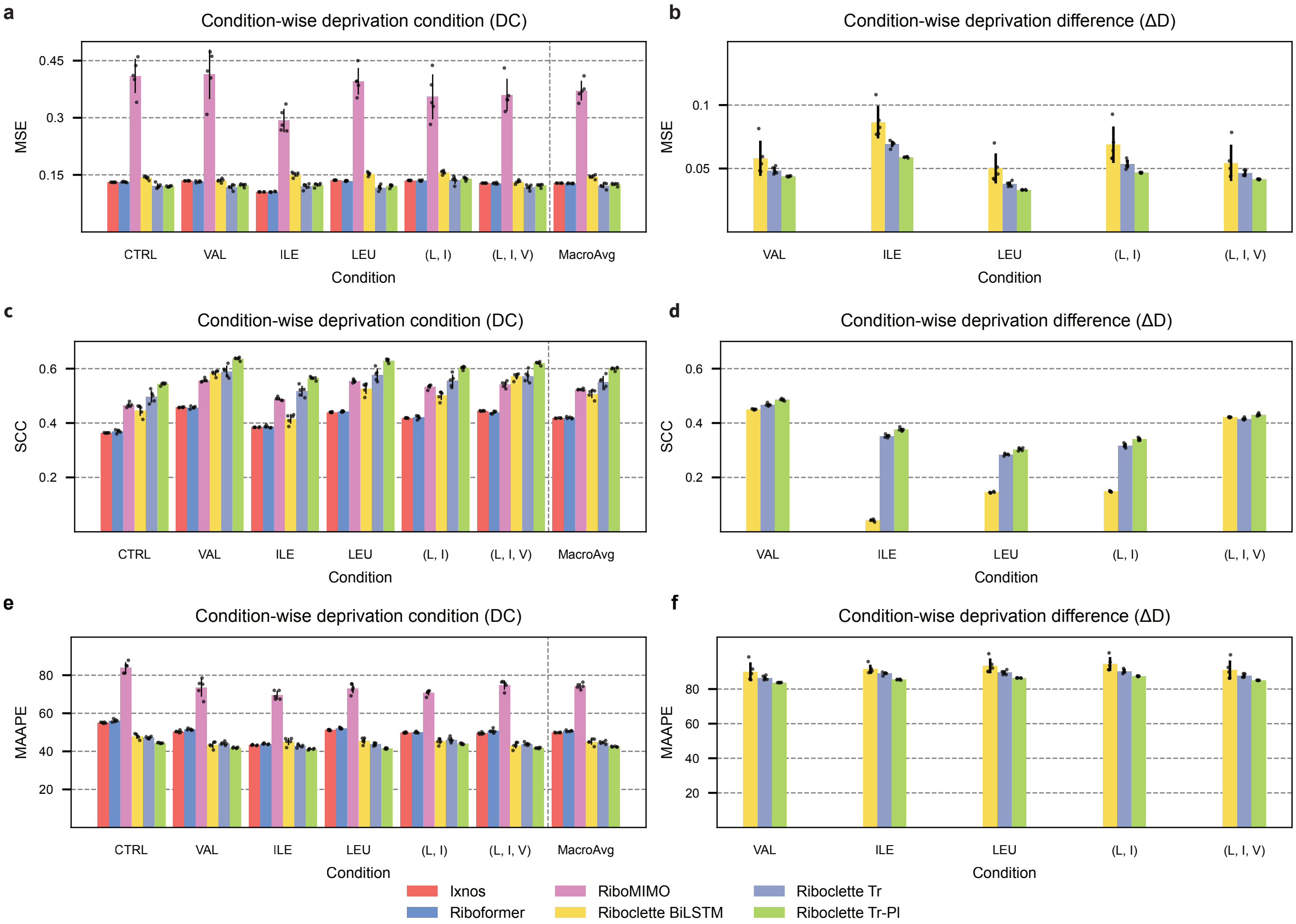

Fig. S2: **Performance evaluation using the Mean Squared Error, Spearman Correlation Coefficient, and Mean Arc-tangent Absolute Percentage Error.** This figure complements figs. 1E and 1F by evaluating the performance of the models with the Mean Squared Error (MSE), Spearman Correlation Coefficient (SCC), and Mean Arc-tangent Absolute Percentage Error (MAAPE). (**a, c, e**) Comparison of the MSE, SCC, and MAAPE, respectively, between the predicted and true RFPs for the three baselines (Iχnos, Riboformer, and RiboMIMO) and the three Riboclette variants across all conditions, along with their macro-average (MacroAvg). The error bars here represent the standard deviation of the respective metrics. (**b, d, f**) Comparison of the MSE, SCC, and MAAPE, respectively, between the predicted and true ΔRFPs for the three Riboclette variants across all conditions. The baselines are excluded as they don't predict ΔRFPs. The error bars here represent the standard deviation of the respective metrics.

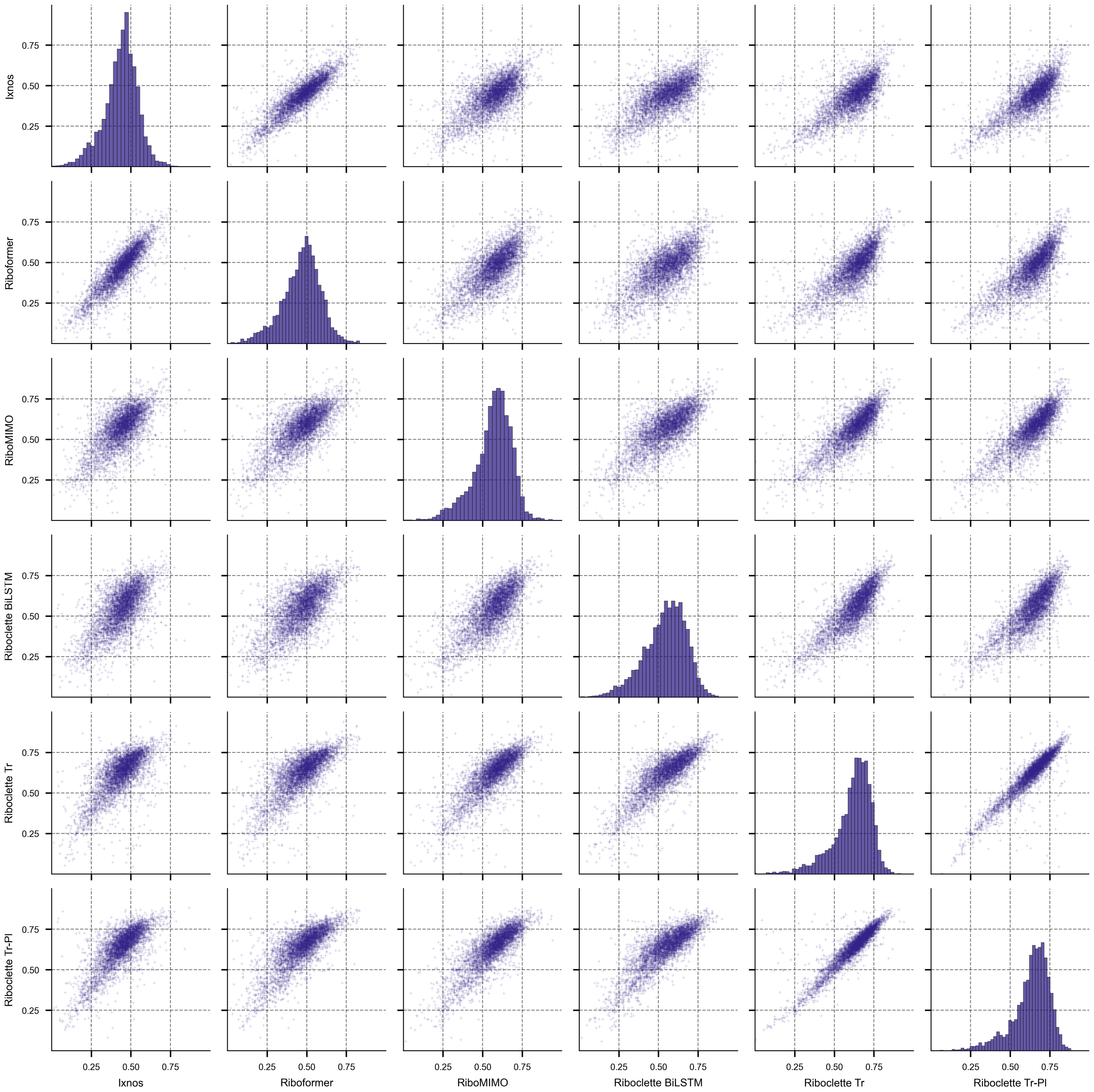

Fig. S3: **Pairwise model performance comparison (PCC).** A comparative analysis of each model's Pearson Correlation Coefficient (PCC) for every gene and condition in the test set. For each model, the best seed was chosen based on the macro average PCC performance across all conditions.

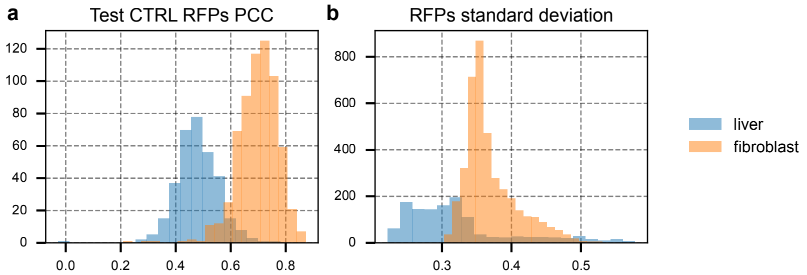

Fig. S4: **Batch effects in the control samples**. Control (CTRL) samples belong to two different *mus musculus* datasets from liver and fibroblast cells (see section 4.1). (**a**) Test set CTRL Ribosome Footprint Profiles (RFPs) PCC distributions of liver and fibroblast samples. (**b**) Standard deviation of all CTRL samples RFPs for liver and fibroblast samples.

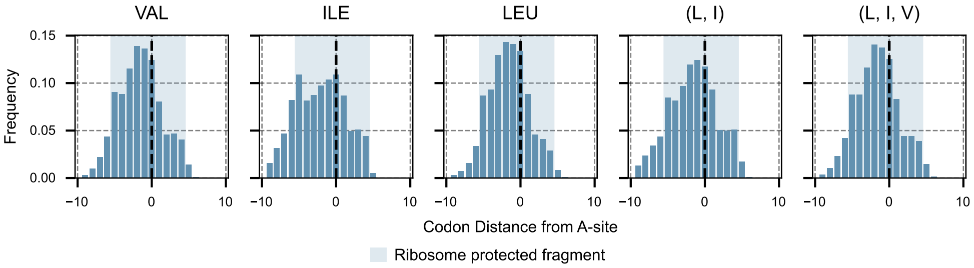

Fig. S5: **Relative position to the A-site distribution of the top 5 codons attribution-wise.** Relative position distribution of the top 5 codons attribution-wise for each deprived condition. In blue, we highlight the ribosome protected fragment.

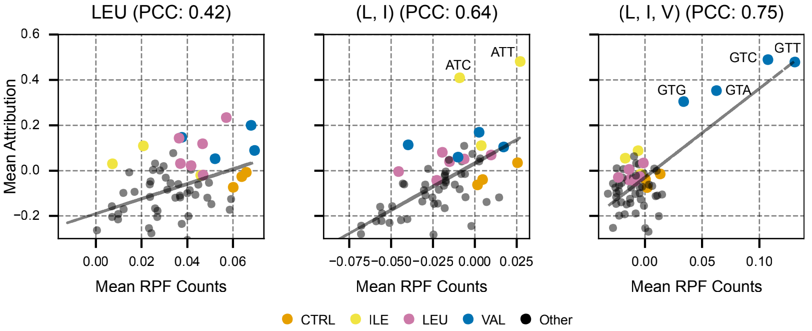

Fig. S6: **Attribution vs. stalling for LEU, (L, I), and (L, I, V).** Correlation between the codon-wise mean attributions across all the dataset and their RFP counts for LEU, (L, I), and (L, I, V). The tagged colored codons represent the top 3 slowest CTRL codons and the codons coding the deprived amino acids.

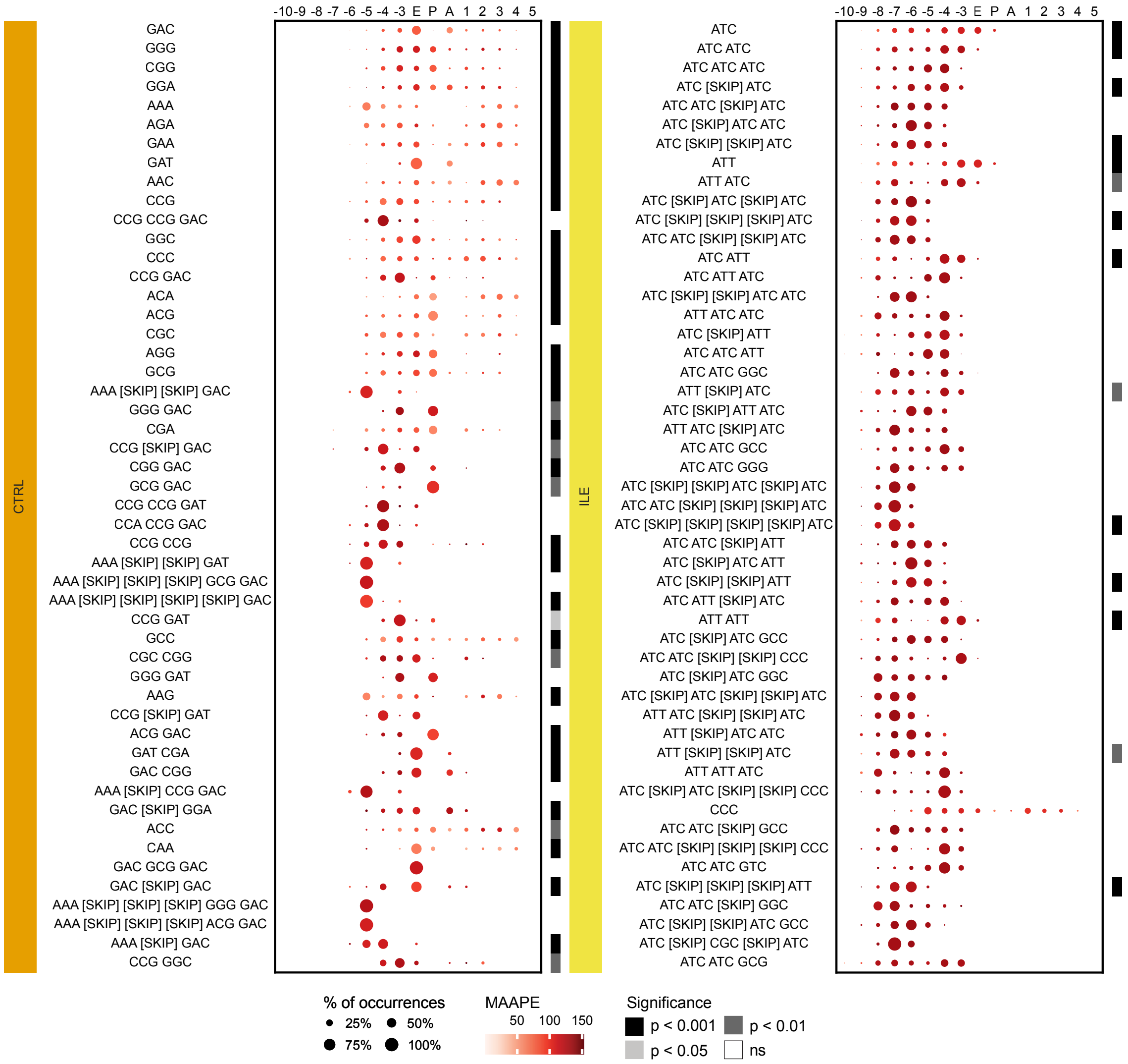

Fig. S7: **Motif Position Heatmap for the Most Frequent Motifs in CTRL and ILE.** Distribution of the top 50 motifs for CTRL and ILE across different relative positions to the A-site. For each motif and position, the occurrence percentage is reported, normalized per motif. The average Mean Arc-tangent Absolute Percentage Error is provided, indicating the relative increase when the given motif is added to the sequence. We also report the adjusted p-values from the Fisher's exact test to assess the statistical significance of motif enrichment in the neighborhoods of RFP peaks compared to any other regions.

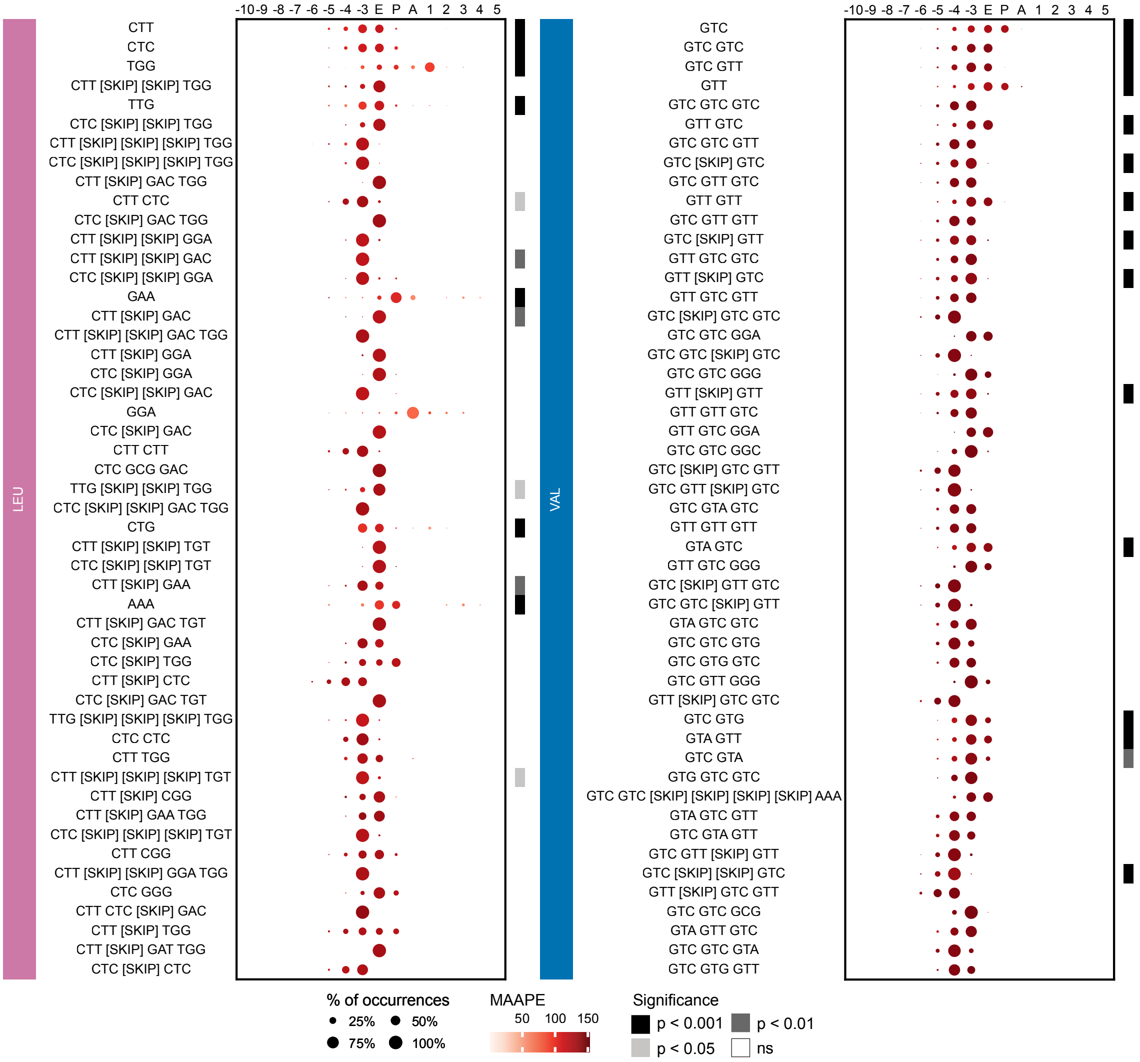

Fig. S8: **Motif Position Heatmap for the Most Frequent Motifs in LEU and VAL.** Distribution of the top 50 motifs for LEU and VAL across different relative positions to the A-site. For each motif and position, the occurrence percentage is reported, normalized per motif. The average Mean Arc-tangent Absolute Percentage Error is provided, indicating the relative increase when the given motif is added to the sequence. We also report the adjusted p-values from the Fisher's exact test to assess the statistical significance of motif enrichment in the neighborhoods of RFP peaks compared to any other regions.

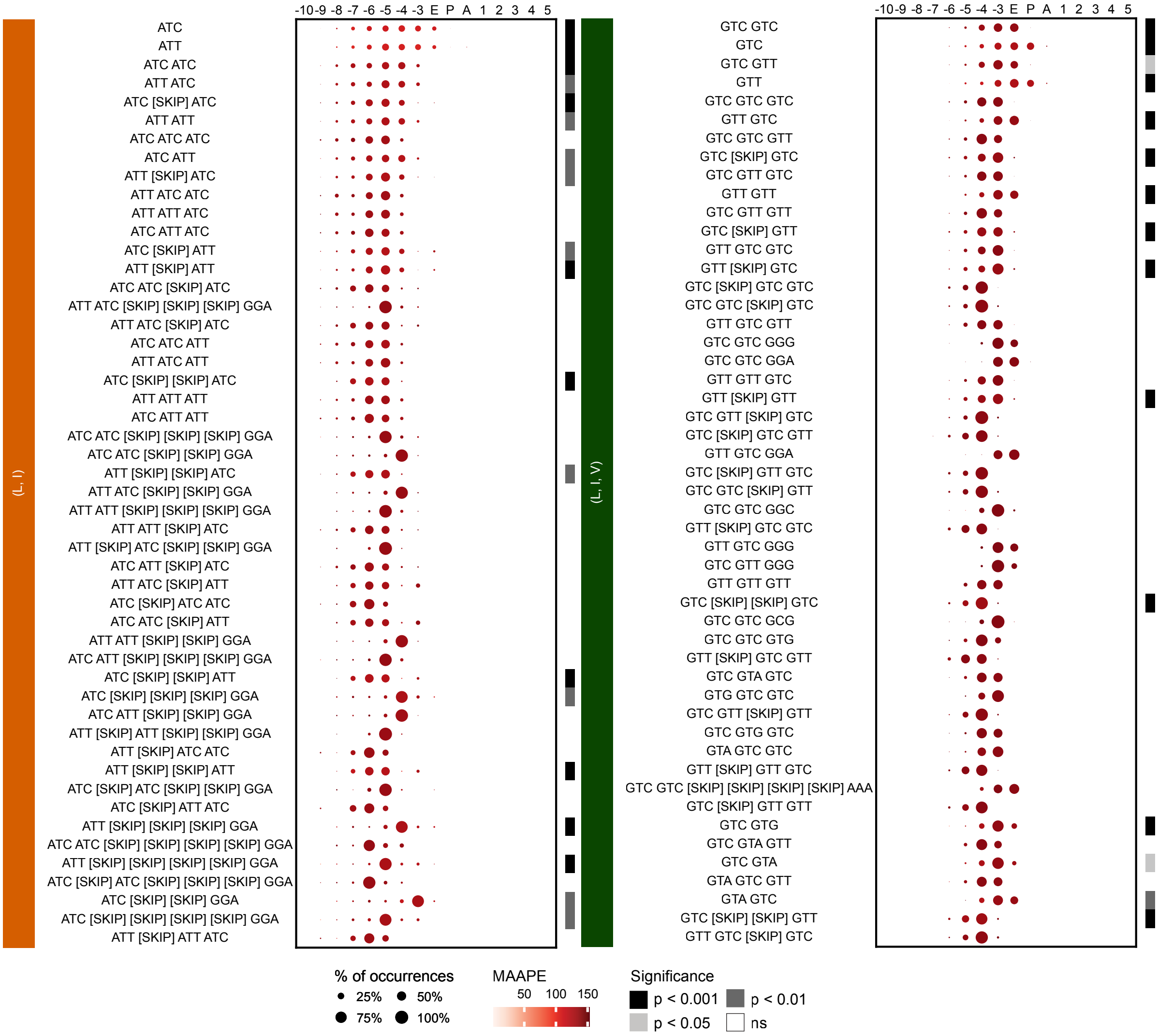

Fig. S9: **Motif Position Heatmap for the Most Frequent Motifs in (L, I) and (L, I, V).** Distribution of the top 50 motifs for (L, I) and (L, I, V) across different relative positions to the A-site. For each motif and position, the occurrence percentage is reported, normalized per motif. The average Mean Arc-tangent Absolute Percentage Error is provided, indicating the relative increase when the given motif is added to the sequence. We also report the adjusted p-values from the Fisher's exact test to assess the statistical significance of motif enrichment in the neighborhoods of RFP peaks compared to any other regions.

Hyperparameter search space and optimal values			
Model Name	Function/Class	Hyperparameter	Value
Riboclette Tr	`transformers.XLNetConfig`	`n_layers`	[1, 2, **3**, 4, 5, 6]
		`d_model`	[64, 128, 256, **512**]
		`n_heads`	[**4**, 8, 16]
		`dropout`	[0.0, **0.1**]
		Batch Size	[**1**, 2, 4, 8, 16]
		Learning Rate	[1e−3, **1**e−**4**, 1e−5]
Riboclette Tr-Pl	`transformers.XLNetConfig`	`n_layers`	[1, 2, 3, 4, 5, **6**]
		`d_model`	[64, 128, 256, **512**]
		`n_heads`	[**4**, 8, 16]
		`dropout`	[0.0, **0.1**]
		Batch Size	[1, **2**, 4, 8, 16]
		Learning Rate	[1e−3, **1**e−**4**, 1e−5]
Riboclette BiLSTM	`torch.nn.LSTM`	`n_layers`	[2, **4**, 6]
		`hidden_size`	[**64**, 128, 256, 512]
		Embedding Size	`hidden_size`
		Batch Size	[**1**, 2, 4, 8, 16]
		Learning Rate	[1e−3, **1**e−**4**, 1e−5]
		Dropout	[**0.0**, 0.1]

Table S2: **Hyperparameter search space and optimal values for the three tuned models.** The tuned hyperparameters include: `n_layers` as the number of layers, `d_model` as the encoder size, `n_heads` as the number of attention heads, `hidden_size` as the size of the hidden layers. For each model, we reported the main hyperparameter function class (from either the `transformer` or `torch` library), moreover, the best hyperparameter is highlighted in bold.

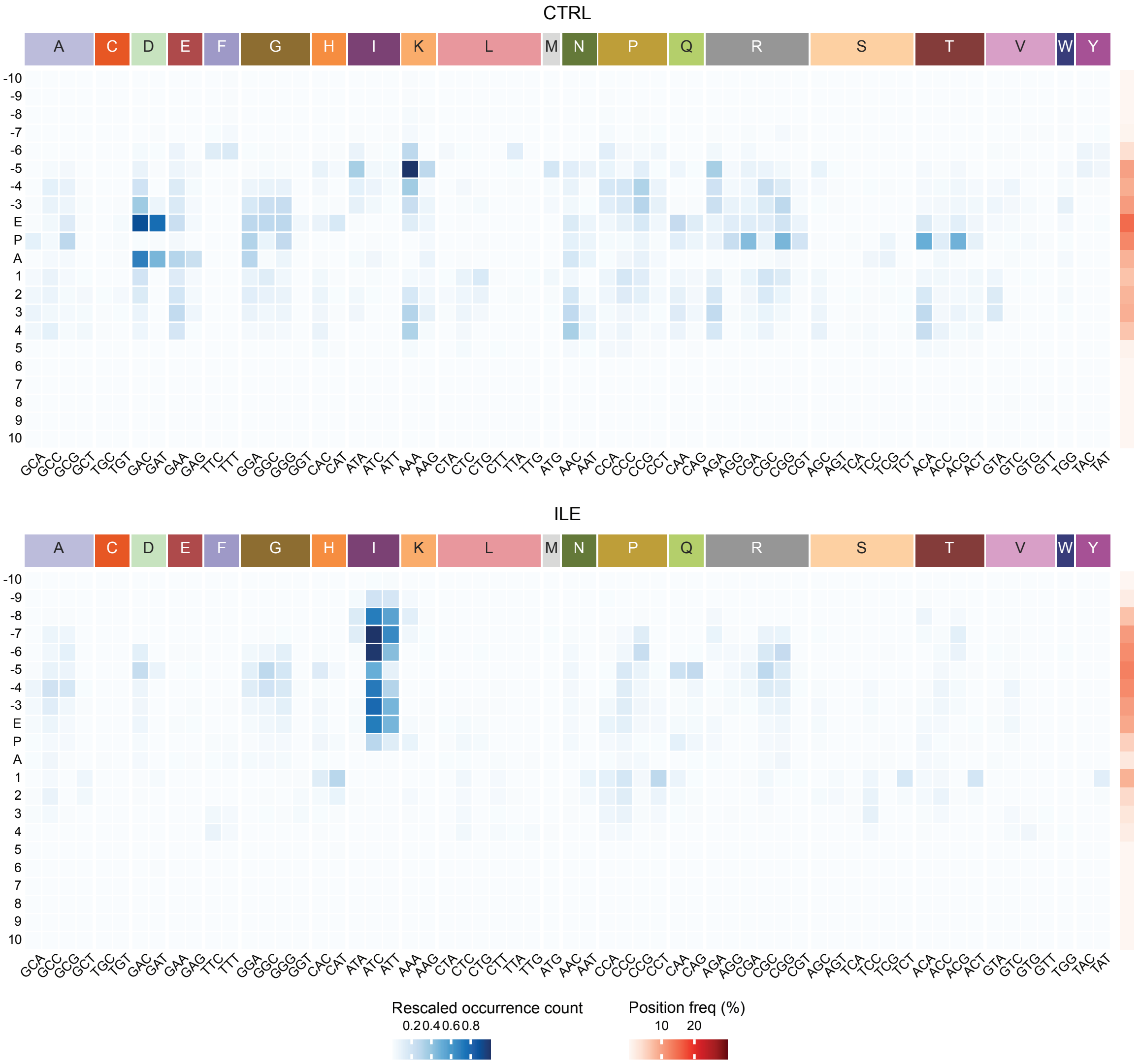

Fig. S10: **Codon occurrence in motifs for the CTRL and ILE conditions.** This visualization represents the distribution of codon positions relative to the A-site of codons within CTRL and ILE motifs. The occurrence count is normalized across the entire heatmap. The position frequency on the right reflects the sum of each row's occurrence count, normalized across all rows.

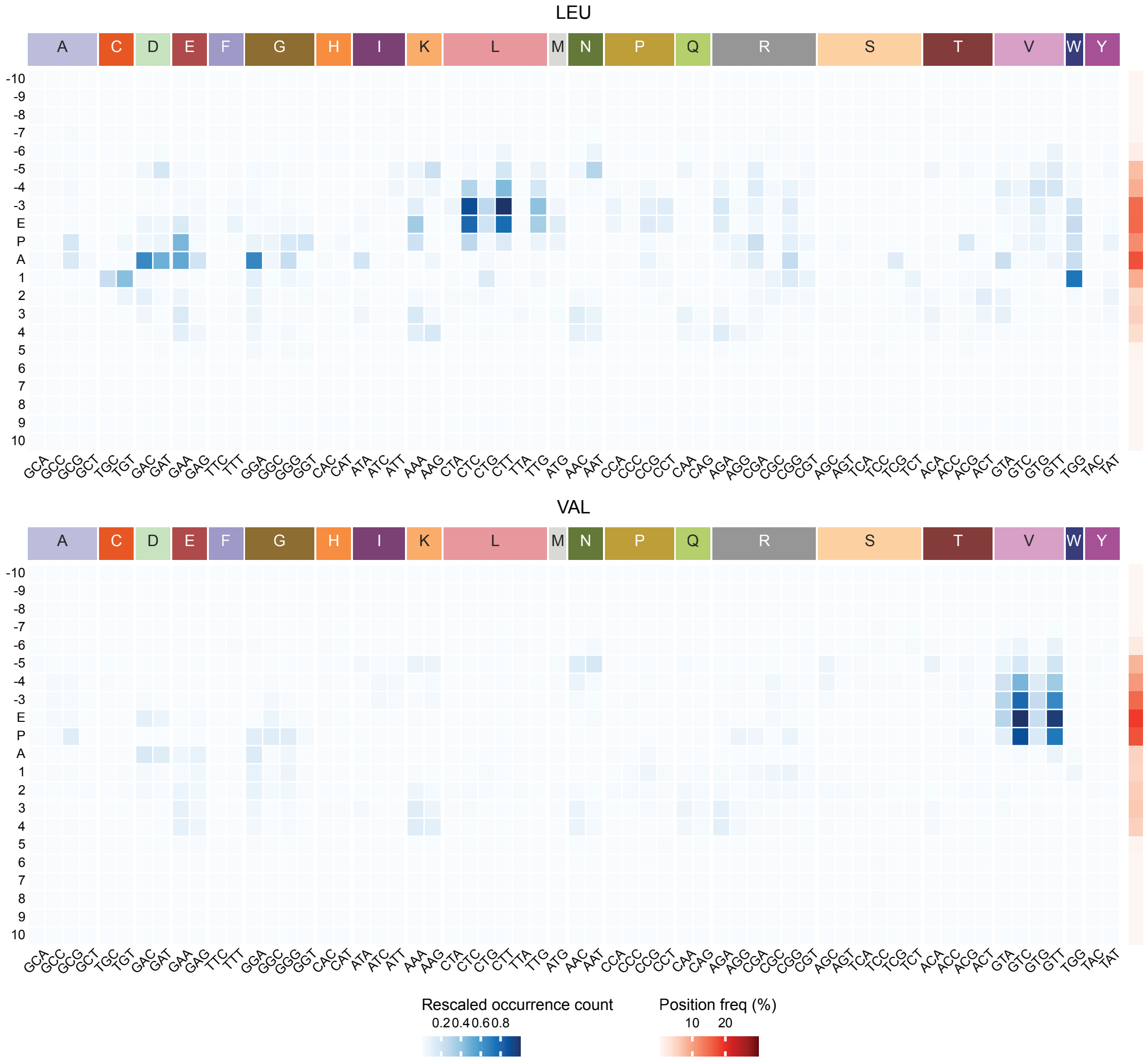

Fig. S11: **Codon occurrence in motifs for the LEU and VAL conditions.** This visualization represents the distribution of codon positions relative to the A-site of codons within LEU and VAL motifs. The occurrence count is normalized across the entire heatmap. The position frequency on the right reflects the sum of each row's occurrence count, normalized across all rows.

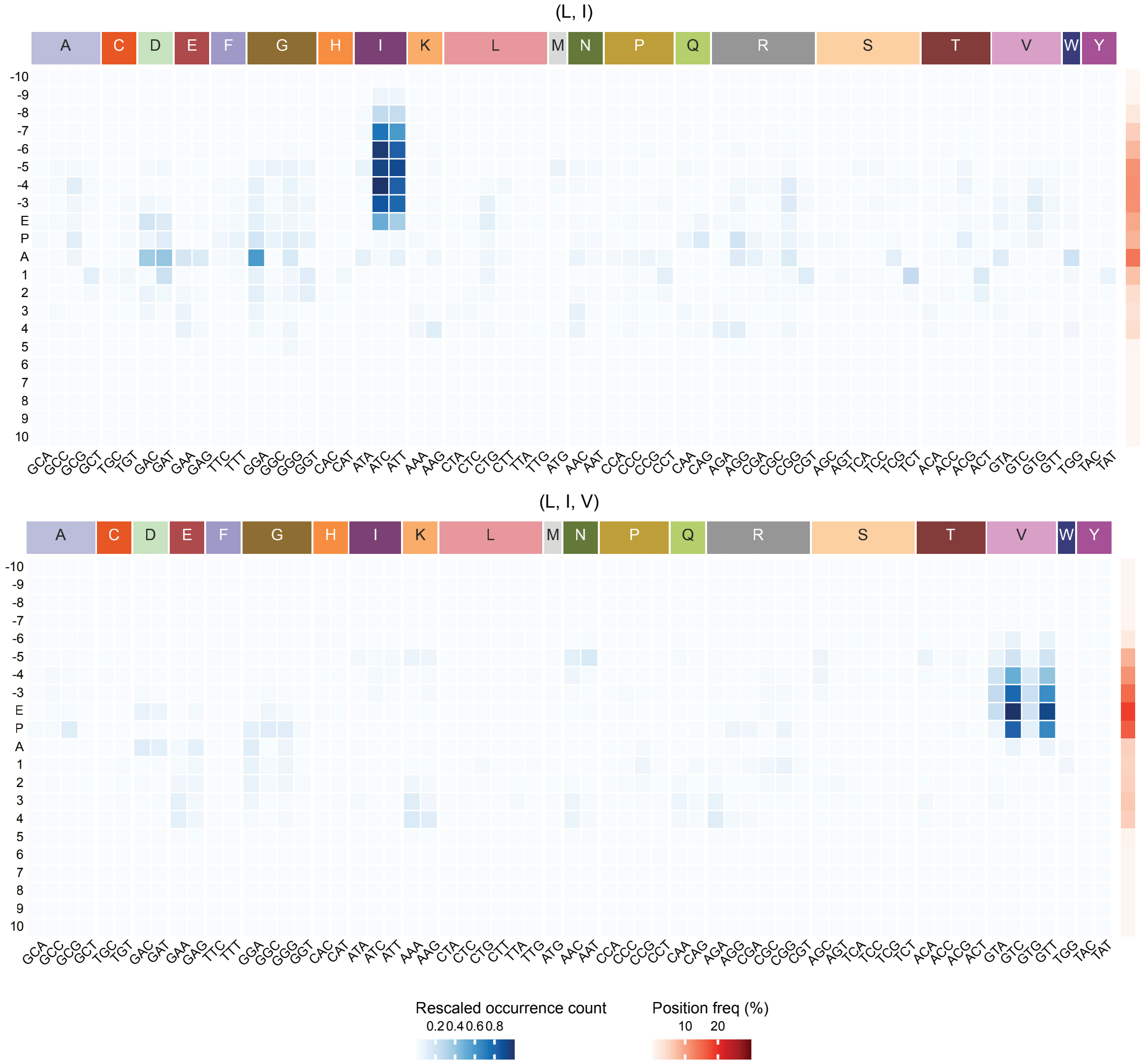

Fig. S12: **Codon occurrence in motifs for the (L,I) and (L,I,V) conditions.** This visualization represents the distribution of codon positions relative to the A-site of codons within (L,I) and (L,I,V) motifs. The occurrence count is normalized across the entire heatmap. The position frequency on the right reflects the sum of each row's occurrence count, normalized across all rows.

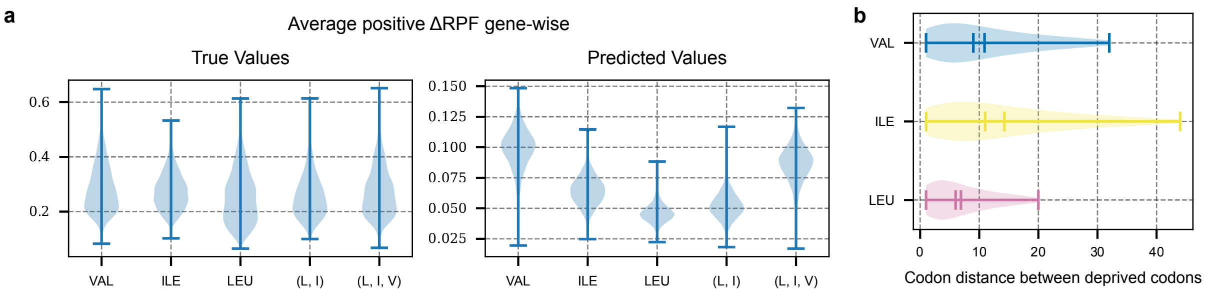

Fig. S13: **Statistics of Deprivation Conditions** (**a**) Distribution of positive values in the Δ ribosome footprint profiles (RFPs), considering only codon positions where ΔRFPs > 0, analyzed separately for each condition. The left plot shows the true pre-processed ΔRFPs, while the right plot presents the predicted values. (**b**) Distribution of distances between codons coding the deprived amino acids across all genes in the dataset.

Supplementary Data 1: Data and statistical test results to validate the significance of each top-50 motif included in figs. S7 to S9. For each motif we reported: (1) the number of times it occurred inside a 10 codon window around a peak (`n_hit_peak`) or (2) in any other position (`n_hit_other`), (3) the number of times it did not occur inside a 10 codon window around a peak (`n_miss_peak`) or (4) in any other position (`n_miss_other`), (5) the unconditional maximum likelihood estimate of the odds ratio (`statistic`), (6) the p-value (`pvalue`), and (7) the adjusted p-value with the Benjamini-Hochberg correction (`pvalue_corrected`) obtained after applying the Fisher's exact test on the counts.

Supplementary References

[1] Liu, T.-Y., Song, Y.S.: Prediction of ribosome footprint profile shapes from transcript sequences. Bioinformatics **32**(12), 183–191 (2016) https://doi.org/10.1093/bioinformatics/btw253

[2] O'Connor, P.B.F., Andreev, D.E., Baranov, P.V.: Comparative survey of the relative impact of mRNA features on local ribosome profiling read density. Nature Communications **7**(1), 12915 (2016) https://doi.org/10.1038/ncomms12915

[3] Zhang, S., Hu, H., Zhou, J., He, X., Jiang, T., Zeng, J.: Analysis of Ribosome Stalling and Translation Elongation Dynamics by Deep Learning. Cell Systems **5**(3), 212–2206 (2017) https://doi.org/10.1016/j.cels.2017.08.004

[4] Tunney, R., McGlincy, N.J., Graham, M.E., Naddaf, N., Pachter, L., Lareau, L.F.: Accurate design of translational output by a neural network model of ribosome distribution. Nature Structural & Molecular Biology **25**(7), 577–582 (2018) https://doi.org/10.1038/s41594-018-0080-2

[5] Cui, H., Hu, H., Zeng, J., Chen, T.: DeepShape: Estimating isoform-level ribosome abundance and distribution with Ribo-seq data. BMC Bioinformatics **20**(24), 678 (2019) https://doi.org/10.1186/s12859-019-3244-0

[6] Hu, H., Liu, X., Xiao, A., Li, Y., Zhang, C., Jiang, T., Zhao, D., Song, S., Zeng, J.: Riboexp: An interpretable reinforcement learning framework for ribosome density modeling. Briefings in Bioinformatics **22**(5), 412 (2021) https://doi.org/10.1093/bib/bbaa412

[7] Tian, T., Li, S., Lang, P., Zhao, D., Zeng, J.: Full-length ribosome density prediction by a multi-input and multi-output model. PLOS Computational Biology **17**(3), 1008842 (2021) https://doi.org/10.1371/journal.pcbi.1008842

[8] Nallapareddy, M.V., Craighero, F., Gobet, C., Naef, F., Vandergheynst, P.: Towards improving full-length ribosome density prediction by bridging sequence and graph-based representations. In: Proceedings of the 19th Machine Learning

in Computational Biology Meeting (2024)

[9] Shao, B., Yan, J., Zhang, J., Liu, L., Chen, Y., Buskirk, A.R.: Riboformer: A deep learning framework for predicting context-dependent translation dynamics. Nature Communications **15**(1), 2011 (2024) https://doi.org/10.1038/s41467-024-46241-8

[10] He, J., Xiong, L., Shi, S., Li, C., Chen, K., Fang, Q., Nan, J., Ding, K., Mao, Y., Boix, C.A., Hu, X., Kellis, M., Li, J., Xiong, X.: Deep learning prediction of ribosome profiling with Translatomer reveals translational regulation and interprets disease variants. Nature Machine Intelligence, 1–16 (2024) https://doi.org/10.1038/s42256-024-00915-6

[11] Aguilar Rangel, M., Stein, K., Frydman, J.: A machine learning approach uncovers principles and determinants of eukaryotic ribosome pausing. Science Advances **10**(42), 0738 (2024) https://doi.org/10.1126/sciadv.ado0738
