# Supplementary material for: Conditional deep learning model reveals translation elongation determinants during amino acid deprivation
Source: Commun Biol. 2025 Nov 26;8:1691. doi: 10.1038/s42003-025-09092-7 (PMC12657957; doi:10.1038/s42003-025-09092-7)
Supplement: Supplementary file 3 — Description of Additional Supplementary Files [file 42003_2025_9092_MOESM3_ESM.pdf]

Description of Additional Supplementary Files

File name- Supplementary Data 1

File description - : Data and statistical test results to validate the significance of each top-50 motif included in figs. S7 to S9. For each motif we reported: (1) the number of times it occurred inside a 10 codon window around a peak (n hit peak) or (2) in any other position (n hit other), (3) the number of times it did not occur inside a 10 codon window around a peak (n miss peak) or (4) in any other position (n miss other), (5) the unconditional maximum likelihood estimate of the odds ratio (statistic), (6) the p-value (pvalue), and (7) the adjusted p-value with the Benjamini-Hochberg correction (pvalue corrected) obtained after applying the Fisher’s exact test on the counts.
